# DNA damage in inhabitants exposed to heavy metals near Hudiara drain, Lahore, Pakistan

**DOI:** 10.1038/s41598-024-58655-x

**Published:** 2024-04-10

**Authors:** Saima Jadoon, Qurban Ali, Adnan Sami, Muhammad Zeeshan Haider, Muhammad Ashfaq, Muhammad Arshad Javed, Mudassar Ali Khan

**Affiliations:** 1 Directorate of Curriculum and Teaching Education, Abbottabad, Pakistan; 2 Institute of Molecular Biology and Biotechnology, University of Lahore, Lahore, Pakistan; 3 Department of Plant Breeding and Genetics, Faculty of Agricultural Sciences, University of the Punjab, P.O BOX. 54590, Lahore, Pakistan; 4 Department of Physiology, Rashid Latif Medical College, Lahore, 54000 Pakistan

**Keywords:** DNA damage, Heavy metals, 8-hydroxyguanosine, MDA, 8-Isoprostane, Ecology, Environmental sciences

## Abstract

The current study was conducted on the inhabitants living in the area adjacent to the Hudiara drain using bore water and vegetables adjacent to the Hudiara drain. Toxic heavy metals badly affect human health because of industrial environmental contamination. Particularly hundreds of millions of individuals globally have faced the consequences of consuming water and food tainted with pollutants. Concentrations of heavy metals in human blood were elevated in Hudiara drainings in Lahore city, Pakistan, due to highly polluted industrial effluents. The study determined the health effects of high levels of heavy metals (Cd, Cu, Zn, Fe, Pb, Ni, Hg, Cr) on residents of the Hudiara draining area, including serum MDA, 8-Isoprostane, 8-hydroxyguanosine, and creatinine levels. An absorption spectrophotometer was used to determine heavy metals in wate water, drinking water, soil, plants and human beings blood sampleas and ELISA kits were used to assess the level of 8-hydroxyguanosine, MDA, 8-Isoprostane in plasma serum creatinine level. Waste water samples, irrigation water samples, drinking water samples, Soil samples, Plants samples and blood specimens of adult of different weights and ages were collected from the polluted area of the Hudiara drain (Laloo and Mohanwal), and control samples were obtained from the unpolluted site Sheiikhpura, 60 km away from the site. Toxic heavy metals in blood damage the cell membrane and DNA structures, increasing the 8-hydroxyguanosine, MDA, creatinine, and 8-Isoprostane. Toxic metals contaminated bore water and vegetables, resulting in increased levels of creatinine, MDA, Isoprostane, and 8-hydroxy-2-guanosine in the blood of inhabitants from the adjacent area Hudiara drain compared to the control group. In addition,. This study also investigated heavy metal concentrations in meat and milk samples from buffaloes, cows, and goats. In meat, cow samples showed the highest Cd, Cu, Fe and Mn concentrations. In milk also, cows exhibited elevated Cu and Fe levels compared to goats. The results highlight species-specific variations in heavy metal accumulation, emphasizing the need for targeted monitoring to address potential health risks. The significant difference between the two groups i.e., the control group and the affected group, in all traits of the respondents (weight, age, heavy metal values MDA, 8-Isoprostane, 8-hydroxyguaniosine, and serum creatinine level). Pearson’s correlation coefficient was calculated. The study has shown that the level of serum MDA, 8-Isoprostane, 8-hydroxyguaniosine, or creatinine has not significantly correlated with age, so it is independent of age. This study has proved that in Pakistan, the selected area of Lahore in the villages of Laloo and Mohanwal, excess of heavy metals in the human body damages the DNA and increases the level of 8-Isoprostane, MDA, creatinine, and 8-hydroxyguaniosine. As a result, National and international cooperation must take major steps to control exposure to heavy metals.

## Introduction

Heavy metals play vital role in biochemical and physiological functions of plants and animals. It is indicated that heavy metals have severe ramifications on cellular organelles and other components^1^. Scientific studies show sewage water causes soil pollution, harming humans and animals. The heavy metals contaminate the drinking water due to improper sewage disposal and municipal waste management. A high quantity of heavy metals is present in industrial wastewater^2^. Heavy metals affect plant growth, easily accumulating in soil and entering the food chain. Essential and non-essential metals exist in our environment^3^. Using wastewater in agriculture results in toxic metal contamination in vegetables^4,5^. The absorption of heavy metals is harmful to our bodies^6^. The Hudiara drain starts from Batala in the Gurdaspur district of India and enters Pakistan from the Laloo village of India to the Hudiara village of Pakistan. The length of the drain in India is 55 km, and 63 km inside Pakistan^7^.

The end junction in Pakistan is the river Ravi. The water is rich in pollutants due to hundreds of industries near the Hudiara drain^8^. The farmers use this wastewater for irrigation purposes, and because of this, there is an excess of heavy metals in vegetables and crops. The heavy metals enter animal bodies (cow, buffalo, goat) through feed and drinking water; when humans eat meat and drink the milk of animals (cow, buffalo, goat), these heavy metals enter the human body^9^. The level of heavy metals in milk (cow, buffalo, goat) is higher than the permissible limit compared to normal in many countries like Pakistan^10^, Egypt^10^, and Nigeria^11^. Meat is the source of protein used for the formation, growth, and repair of tissues^12^. Heavy metals toxicity in meat causes toxic effects on human health^10^.

The bioaccumulation of heavy metals in the food chain causes a serious health threat to human beings^13^. Metals directly affect on mental development and cause cancer^13^. Drinking polluted water and using wastewater for irrigation of vegetables and crops transfer heavy metals to the meat of animals and contaminate human feed^14^ Trace elements may cause oxidative DNA damage in living organisms, particularly humans. Biomarkers are considered a vital tool for exposure identification, and their use may provide important information^15^. In terms of Biochemistry, Biomarkers are observable endpoints in the continuity of events leading from exposure of environmental agents to diseases^16–18^. Once validated through laboratory tests and studies, biomarkers can provide direct measures of the actual effect of chemicals upon living organisms in the field, thereby overcoming large areas of uncertainty expressed in standard risk assessment^19^. 8-OHdG serum level measurement may be a valuable marker for biomonitoring in the case of mixed occupational exposure to toxic metals and increased cancer risk^20^.

The present study aimed to determine the concentrations of heavy metals like iron, zinc, copper, cadmium, arsenic, lead, and mercury in irrigation water, drinking water, soil, vegetables, milk, beef, goat meat, and human serum samples. This study selected and also used three biomarkers 8-hydroxy-2-deoxyguanosine, malondialdehyde (MDA), and 8-isoprostane, as three bioindicators to demonstrate the effect of heavy metals on human health in the vicinity of Hudiara drain.

## Material and method

The experimental data collection followed all the rules and regulations at the Institute of Molecular Biology and Biotechnology, The University of Lahore, Lahore, Pakistan. Permissions from the authorities were obtained as required. It has been confirmed that the plant samples were collected and used while complied with relevant institutional, national, and international guidelines and legislation with appropriate permissions from authorities of the Local Government authorities of District Lahore and Sheikpura, and the Institute of Molecular Biology and Biotechnology, The University of Lahore, Lahore, Pakistan. Ethical Review Committee of the Institute of Molecular Biology and Biotechnology, University of Lahore, Lahore, Pakistan, gave the ethical approval, with reference number 2018010120. It has also confirmed that informed consent was obtained from all subjects and/or their legal guardian(s). It has also been confirmed that the study was reported following ARRIVE guidelines.

### Study area

**Figure 1:**
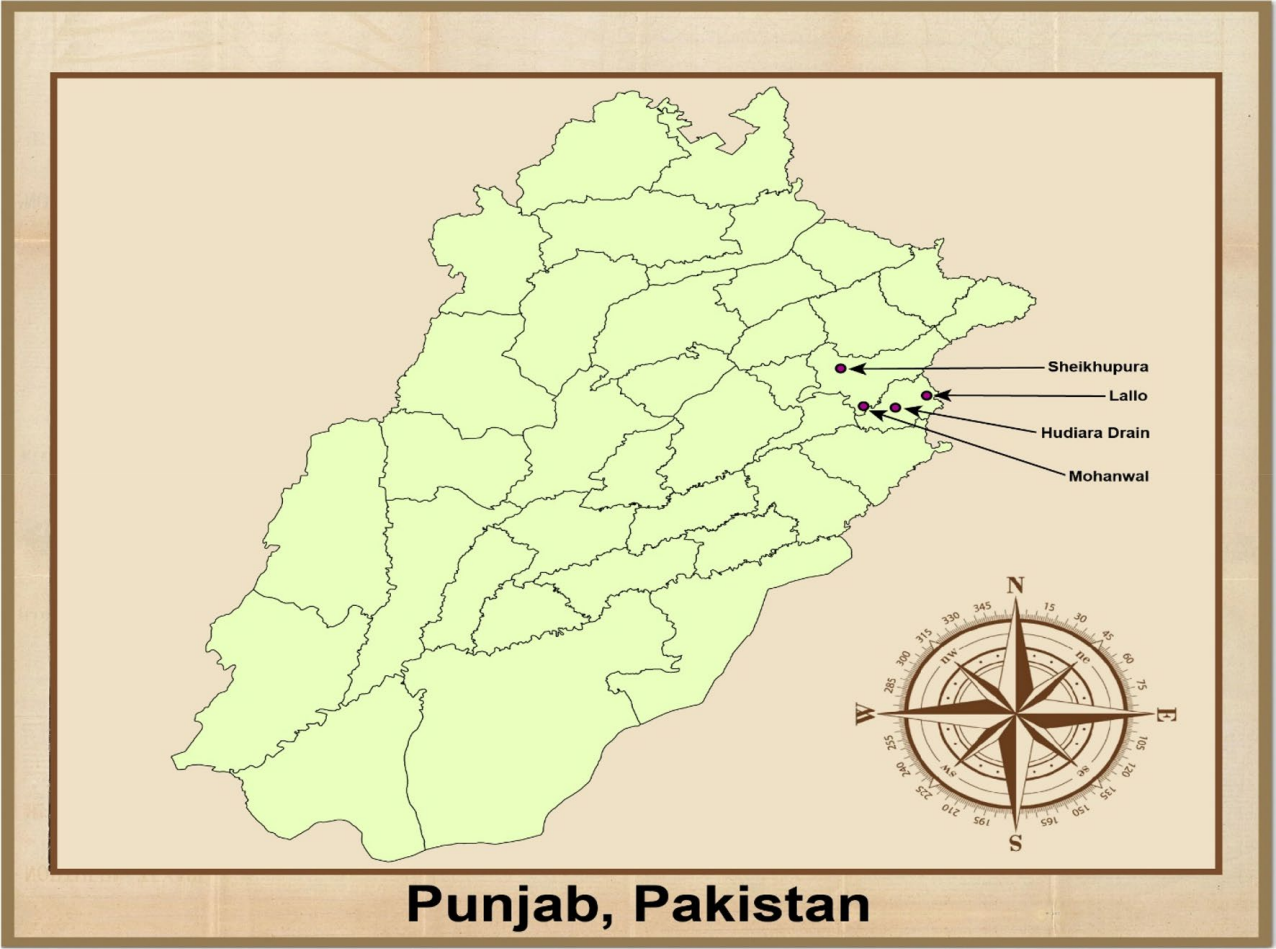
Location map for collection of samples.

The study area is Hudiara, located in the Lahore area in Punjab of Pakistan, with latitude and longitude 31^0^ 2327 N 74^0^ 1427 E. The control area (Sheikpura) was located 60 km away from Hudiara Drain Lahore, Punjab, Pakistan, with latitude and longitude 31^0^766 N 73^0^984 E (Figure 1). Herbaceous are present for grazing animals. The main grain crops of the area are wheat, rice, and vegetables: carrots, cauliflower, turnip, Mustard, spinach, beet, cabbage, garlic, and onion. The study area of this research project was the Laloo and Mohanwal villages of Lahore.

We followed the rules when collecting plant samples in Laloo and Mohanwal villages of Lahore. We got the green light from the local authorities and the Institute of Molecular Biology and Biotechnology at the University of Lahore, Pakistan. Our methods align with ARRIVE guidelines, and the Ethical Review Committee at the institute approved our work with reference number 2018010120. It has also confirmed that informed consent was obtained from all subjects and/or their legal guardian(s).

### Samples collection

Wastewater samples were collected from the surface of stagnant water in the Hudiara drain. Samples of irrigation water were collected from the channels used by farmers in the fields after pumping water. The vegetables were collected from the same field irrigating Hudiara drain water. The polyethylene bottle container was washed with detergent, rinsed with distilled water, and pre-treated with 20% nitric acid (HNO_3_), then finally rinsed through with deionized water, followed by drying in an oven. Ten samples of each vegetable and cereal were collected from the adjacent area of the Hudiara drain (Laloo and Mohanwal). Ten samples of each plant's edible parts (cereals and vegetables) were collected. The rice samples (*Oryza sativa*) and leaves of fodder crop samples were collected in the summer of 2017. The Wheat (*Triticum aestivum*), spinach (*Spinacia oleracea*), onion (*Allium cepa*), potato (*Solanum tuberosum*), cabbage (*Brassica oleracea* var. capitata), cauliflower (*Brassica oleracea* var. botrytis), mustard (*Brassica campestris*), turnip (Brassica *rapa* subsp. Rapa), beet (*Beta vulgaris* subsp), garlic (*Allium sativum*) and carrot (*Daucus carota*) in 2018 winter.

Fifty drinking water samples (250 ml) were collected randomly from tape and Laloo and Mohanwal village wells adjacent to Hudiara drain. Fifty soil samples (250 g) were collected randomly from different places adjacent to Hudiara drain and agriculture fields with a 5-10 cm depth. Ten samples of milk (Goat, Cow, and Buffalo) and ten samples of (buffalo, cow, and goat) meat (buffalo, cow, and goat) were collected from the Laloo and Mohanwal adjacent to the Hudiara drain. Three hundred human blood samples were collected from the Median Cubital vein of adults who are inhabitants of Hudiara drain in 2017–2018. These samples were analyzed in the laboratory of Institute of Molecular Biology and Biotechnology, University of Lahore, Pakistan to determine heavy metals and again verified at Comsat University Abbottabad. The same sample size of control samples of water used for irrigating, soil, vegetables, cereals, meat, milk, and human blood was collected 60 km from Hudiara drain. Anti-coagulant (EDTA) was added for whole blood analysis and placed in the refrigerator. The blood and serum were separated by using a centrifugation process. The samples were digested for further analysis of metals. This study has two groups: a control group of drinking water, irrigation water, plants, animals, and human beings; the second is the affected heavy metals group. One group is the affected inhabitants (Demographic data: supplementary material table 1) near Hudiara Drain, and the second group is the control group 60 km (Sheikhpura) away from Hudiara Drain.

### Methods for standard solution preparation

Heavy metal analysis used standard solutions for drinking water, wastewater, plants, soil, milk, meat, and blood samples. The stock of different heavy metals in nitrate and sulfate form has a concentration of 1000 ppm^21^. The standard solutions for all the heavy metals under study were prepared in three to five different concentrations to obtain a calibration curve by diluting the stock standard solution of a concentration of 1000 ppm^22^.

### Digestion methods

Digestion is necessary before applying the solution to the atomic absorption instrument. Drinking water is digested for application on an atomic absorption spectrophotometer^23^. Different acid mixtures were used (${HNO}_{3}$ ,${H}_{2}{SO}_{4}$, HCl, HF in the ratio of 2:1:1:1) for digestion^24^. The digestion of wastewater samples is carried out by adding aqua regia^25^. The plant samples were dried and digested^26^. The raw milk samples were collected from buffalo, goats, and cows of the adjacent inhabitants of Hudiara drain in washed polyethylene containers and digested^27^. Fresh meat samples of cow (beef), buffalo (beef), and goat (mutton) were collected from the adjacent local meat shops of Hudiara drain, digested, and applied for further analysis of heavy metals^28^. The digestion of blood samples was carried out by taking ${2cm}^{3}$ of blood (3000 rpm for 10 min)^29^. The EIA standard 8-Isoprostane ( Cat# EK7123) was used for quantitative determination^30^. The 8-hydroxy-2’-deoxyguanosine (Cat # MBS 267,161) was quantitatively determined in serum by using ELISA kit by Glory Science Co. Ltd. USA. For MDA (Cat# 90357) centrifuged at the speed of 3000 *rpm* for 600 s. After centrifugation, it separates the top layer and measures the absorbance of the solution at 532 nm^31^. The Cayman Chemical USA creatinine kit (Cat# 700460) is used by using the Jaffe Method for the determination of creatinine in blood serum levels in adult males. The absorbance was measured at 490 nm^31^.

### Statistical analysis

Statistical paired comparison analysis is used for the comparison of selected heavy metals in stagnant and running water of Huidara Drain, heavy metals in drinking water are shown by t-statistic and p-value for significant difference where ANOVA F-test is used for a statistical difference of control and affected group. Concentration of heavy metals in vegetables, meat and milk is represented in graphical form. The statistical analysis in tabulated and graphical form is done by using SPSS Statistic 29.

### Ethical approval

The ethical approval was given by Ethical Review Committee of Institute of Molecular Biology and Biotechnology, University of Lahore, Lahore, Pakistan with references number 2018010120. It has also confirming that informed consent was obtained from all subjects and/or their legal guardian(s).

## Results

### Heavy metal concentrations in hudiara drain

In this study, Table 1 provides a comparative analysis of the concentrations of selected heavy metals (Fe, Cu, Mn, Ni, Cr, Cd, Pb, Zn) in both stagnant and running water within the Hudiara Drain. Understanding the variations in heavy metal levels in different water conditions is essential for assessing this important waterway's environmental impact and potential risks. Table 1,2,3 offers insights concentration of heavy metals in running (irrigation) and stagnant water and shows the concentration of heavy metals high in stagnant water as compare to running water.

**Table 1:** Quantity of selected heavy metals (Fe, Cu, Mn, Ni, Cr, Cd, Pb, Zn) in stagnant and running water of Hudiara drain in vicinity of Lahore.

Heavy metals in stagnant water of hudiara drain water	Mean	Std. deviation	Heavy metals inirrigating hudiara drain water	Mean	Std. deviation
Fe (mg/l)	818.46	84.458	Fe (mg/l)	423.4100	108.25304
Cu (mg/l)	810.68	108.475	Cu (mg/l)	399.4976	79.35818
Mn (mg/l)	83.32	13.367	Mn (mg/l)	28.44	8.825
Ni (mg/l)	215.04	29.802	Ni (mg/l)	134.38	43.130
Cr (mg/l)	337.96	77.448	Cr (mg/l)	161.40	76.648
Cd (mg/l)	428.78	94.477	Cd (mg/l)	264.06	86.145
Pb (mg/l)	586.50	69.740	Pb (mg/l)	367.18	50.180
Zn (mg/l)	396.46	51.393	Zn (mg/l)	280.82	40.724

**Table 2:** Comparison of concentration of selected heavy metals (Fe, Cu, Mn, Ni, Cr, Cd, Pb, Zn) in stagnant and running water of Hudiara drain in vicinity of Lahore.

Paired comparison analysis		N	Mean	Std. deviation	t-Statistics	P-Values
Pair 1	Fe mg/l in stagnant water–Fe mg/l in hudiara drain water	50	395.05000	131.72349	21.207	0.000
Pair 2	Cu mg/l in stagnant water–Cu mg/l in hudiara drain water	50	411.18240	137.07649	21.211	0.0000
Pair 3	Mn mg/l in stagnant water–Mn mg/l in hudiara drain water	50	54.880	16.473	23.557	0.000
Pair 4	Ni mg/l in stagnant water–Ni mg/l in hudiara drain water	50	80.660	49.933	11.422	0.000
Pair 5	Cr mg/l in stagnant water–Cr mg/l in hudiara drain water	50	176.560	93.018	13.422	0.000
Pair 6	Cd mg/l in stagnant water–Cd mg/l in hudiara drain water	50	164.720	102.243	11.392	0.000
Pair 7	Pb mg/l in stagnant water–Pb mg/l in hudiara drain water	50	219.320	83.392	18.597	0.000
Pair 8	Zn mg/l in stagnant water–Zn mg/l in hudiara drain water	50	115.640	65.568	12.471	0.000

**Table 3:** Quantity of selected heavy metals in soil (Fe, Cu, Zn, Pb, Cd, Cr, Ni and Mn) in control and affected soil samples in vicinity of Hudiara drain, Lahore.

Heavy Metals in soil (mg/kg)	Group	N	Mean	Std. deviation	F-statistics	P-values
Fe	Control	50	46.0120	8.10481	275.163	0.000
	Affected	50	696.2400	277.05754		
Cu	Control	50	6.8260	1.16668	182.411	0.000
	Affected	50	10.6380	1.61926		
Zn	Control	50	3.0660	1.00663	732.818	0.000
	Affected	50	22.8826	5.07744		
Pb	Control	50	0.9094	0.16935	116.942	0.000
	Affected	50	5.5438	3.02562		
Cd	Control	50	0.0051	0.00188	519.831	0.000
	Affected	50	1.0972	0.33868		
Cr	Control	50	0.0678	0.01389	65.677	0.000
	Affected	50	2.6236	2.22995		
Ni	Control	50	0.4118	0.12586	1272.874	0.000
	Affected	50	1.0580	0.02373		
Mn	Control	50	0.6660	0.35584	397.128	0.000
	Affected	50	5.8184	1.79326		

### Quantity of selected heavy metals in soil

Table 3 offers insights into the contamination levels of these heavy metals in the soil, shedding light on the environmental impact and potential risks associated with soil quality near the drain.

### Heavy metals in fodder crop leaves and wastewater of Hudiara drain

Table 3 provides valuable information on the concentrations of selected heavy metals in the leaves of fodder crops and wastewater samples collected from Hudiara Drain. This data is critical for assessing potential contamination and risks associated with the consumption of crops grown in this area, as well as the environmental impact of heavy metal presence in the wastewater of Hudiara Drain.

### Concentration of heavy metals in drinking water

Table 4 presents crucial information regarding the concentrations of various heavy metals in the drinking water of the areas adjacent to the Hudiara Drain. Understanding the levels of these heavy metals in the drinking water is vital for assessing the potential health and environmental impacts in the vicinity of Hudiara Drain.

**Table 4:** Concentration of selected heavy metals (Fe, Cu, Mn, Ni, Cr, Cd, Pb, Zn) in fodder crop leaves and wastewater of Hudiara drain.

Paired comparison analysis		N	Mean	Std. deviation	t-Statistics	P-Values
Pair 1	Fe mg/l in stagnant water–Fe mg/l in hudiara drain water	10	395.05000	131.72349	21.207	0.000
Pair 2	Cu mg/l in stagnant water–Cu mg/l in hudiara drain water	10	411.18240	137.07649	21.211	0.000
Pair 3	Mn mg/l in stagnant water–Mn mg/l in hudiara drain water	10	54.880	16.473	23.557	0.000
Pair 4	Ni mg/l in stagnant water–Ni mg/l in hudiara drain water	10	80.660	49.933	11.422	0.000
Pair 5	Cr mg/l in Stagnant Water–Cr mg/l in hudiara drain water	10	176.560	93.018	13.422	0.000
Pair 6	Cd mg/l in stagnant water–Cd mg/l in hudiara drain Water	10	164.720	102.243	11.392	0.000
Pair 7	Pb mg/l in stagnant water–Pb mg/l in hudiara drain water	10	219.320	83.392	18.597	0.000
Pair 8	Zn mg/l in stagnant water–Zn mg/l in hudiara drain water	10	115.640	65.568	12.471	0.000

### Level of heavy metals and biomarkers in blood serum of males

Table 5 provides comprehensive data on the levels of various heavy metals along with selected biomarkers such as 8-Isoprostane, 8-Hydroxy-2-Deoxyguanosine, Malondialdehyde (MDA), and Creatinine, in the blood serum of two groups—a control group and an affected group of males. This table is a crucial resource for assessing the potential health impacts and biomarker variations in individuals residing near the source of heavy metal exposure.

**Table 5:** Concentration of heavy metals (Cd. Co, Cu, Cr, Fe, Hg, Mn, Ni, Pb, Zn) in drinking water of adjoining area of Hudiara drain.

Heavy metals	N	Mean	Std. deviation	Test value	Mean difference	t-statistics	P-value
Zn (mg/l)	50	0.7996	0.53207	0	0.79960	10.626	0.000
Fe (mg/l)	50	1.4662	1.51325	0	1.46620	6.851	0.000
Cu (mg/l)	50	0.9578	0.86190	0	0.95780	7.858	0.000
Mn (mg/l)	50	0.3084	0.18019	0	0.30840	12.103	0.000
Cd (mg/l)	50	0.0061	0.00211	0.005	0.00110	3.683	0.001
Pb (mg/l)	50	0.0318	0.01804	0.01	0.02180	8.547	0.000
Cr (mg/)l	50	0.2780	0.16449	0.1	0.17800	7.652	0.000
Ni (mg/l)	50	0.0712	0.01452	0.07	0.00120	0.584	0.562

### Concentration of heavy metals in cereals and vegetables

**Figure 2:**
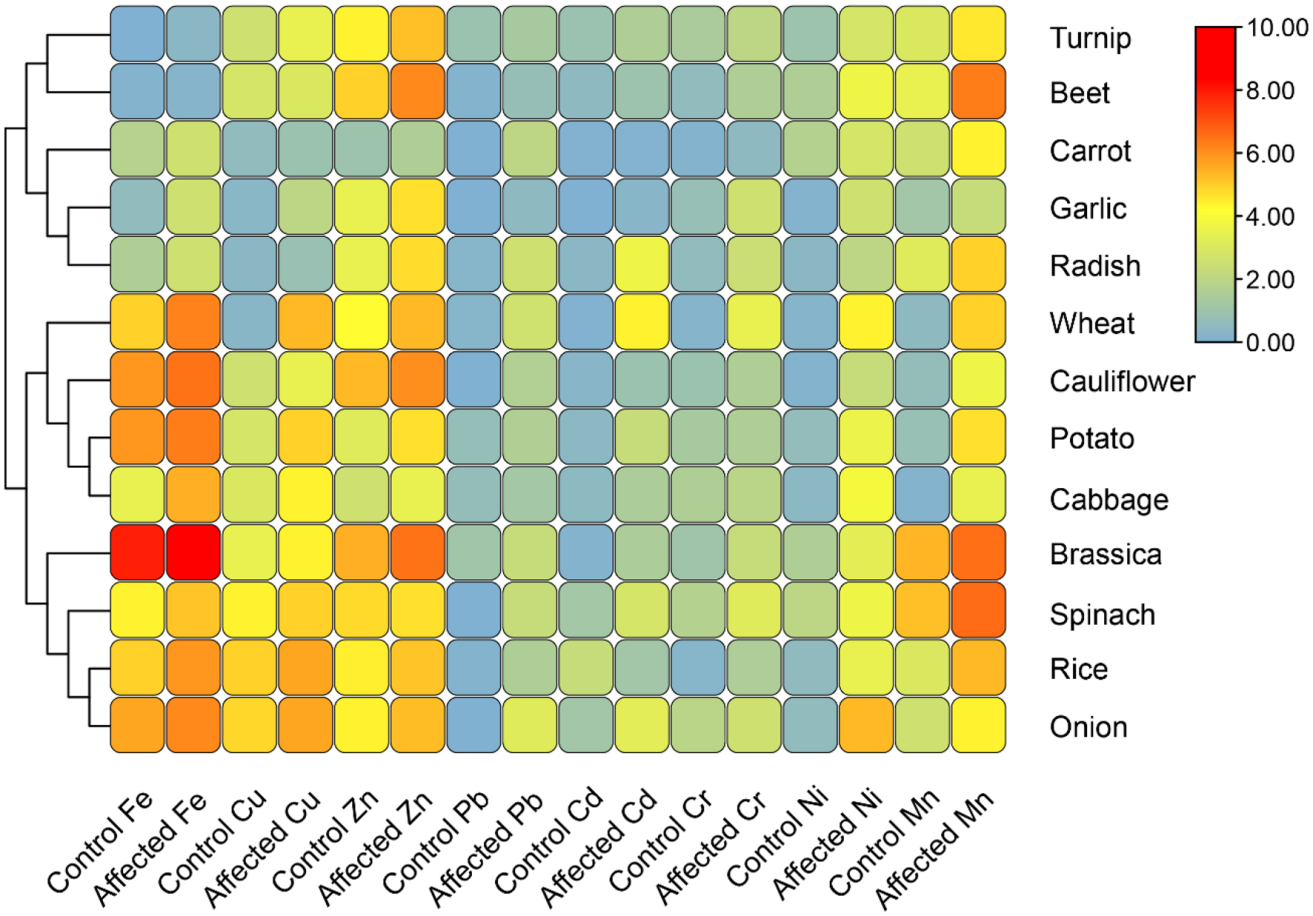
Concentration of Fe, Cu, Mn, Ni, Cr, Cd, Pb and Zn in Cereals and Vegetables of control and affected group of Hudiara drain vicinity in Lahore.

Our research and investigation have revealed significant differences in the heavy metal content of various vegetables and cereals between the control and affected groups (Fig. 2). In the case of iron (Fe), concentrations in affected wheat, spinach, rice, onion, and potato were notably higher than their respective controls, with values ranging from 0.16 to 340 in the simulated samples. Similarly, affected cabbage, Brassica, Turnip, Beet, Garlic, Carrot, and Radish also exhibited elevated iron concentrations. Copper (Cu) levels significantly increased in affected wheat, spinach, rice, onion, and several other samples. Zinc (Zn) content saw substantial elevation in affected wheat, spinach, rice, onion, and different samples, with concentrations ranging from 2 to 90 Table 6.

**Table 6:** Level of heavy metals (Cd. Co, Cu, Cr, Fe, Hg, Mn, Ni, Pb, Zn) and biomarkers (8- isoprostane, 8-Hydroxy-2-Deoxyguaniosine, MDA, Creatinine) in blood serum of control and affected group of males.

Variables		Mean ± Std. deviation	ANOVA F-statistics	(P-value)
Weight Kg	Control group	52.07 ± 6.703	150.226	0.000
	Affected group	62.16 ± 12.585		
Age Years	Control group	41.59 ± 8.173	1.017	0.314
	Affected group	42.56 ± 14.519		
8-Isoprostane pg/ml	Control group	1.2045 ± 0.49960	27,380.978	0.000
	Affected group	49.6800 ± 5.04943		
8-hydroxy-2-deoxyguaniosine mg/ml	Control group	0.2099 ± 0.02475	20,957.601	0.000
	Affected group	1.3893 ± 0.13891		
MDA nmol/ml	Control group	1.8011 ± 0.60081	763.537	0.000
	Affected group	3.8609 ± 1.14285		
Creatinine mg/dl	Control group	0.6481 ± 0.11428	3571.839	0.000
	Affected group	1.7653 ± 0.30292		
Concentration of Cd in human blood µg/l	Control group	33.8128 ± 26.23737	300.700	0.000
	Affected group	372.8357 ± 337.60983		
Co µg/l	Control group	243.30 ± 143.807	1045.855	0.000
	Affected group	536.30 ± 62.810		
Cu µg/l	Control group	1650.30 ± 893.352	96.204	0.000
	Affected group	1108.40 ± 343.00		
Cr µg/l	Control group	0.1385 ± 0.10129	2168.731	0.000
	Affected group	2.7732 ± 0.97467		
Fe µg/l	Control group	0.07805 ± 0.052758	3.089	0.079
	Affected group	0.22379 ± 1.435382		
Hg µg/l	Control group	0.4394 ± 0.34586	1294.195	0.000
	affected group	4.5963 ± 1.97130		
Cd µg/l	Control group	608.37 ± 102.580	3369.421	0.000
	affected group	1172.10 ± 133.314		
Ni µg/l	Control group	0.2277 ± 0.10639	310.909	0.000
	affected group	1.4972 ± 1.24251		
Pb µg/l	Control group	48.92 ± 29.598	785.124	0.000
	Affected group	1315.21 ± 782.195		
Zn µg/l	Control group	156.09 ± 48.918	401.651	0.000
	Affected group	282.77 ± 97.942		

Lead (Pb) concentrations were notably increased in several affected samples, with values reaching as high as 8 in onion. Cadmium (Cd) levels also showed a considerable increase in some affected samples, with values up to 20 in wheat. Chromium (Cr) was elevated in affected wheat (10), spinach (8), onion (5) and garlic (5) samples. Nickel (Ni) was significantly higher in various affected samples, reaching 40 in onion. Finally, manganese (Mn) levels were elevated in affected spinach, with values as high as 98 others were Brassica (94), beet (80) and rice (40). These results indicate the substantial impact of heavy metal contamination on the composition of these vegetables and cereals, raising significant concerns about their safety for consumption.

### Concentration of heavy metal (mg/kg) in buffalo, cow and goat meat

**Figure 3:**
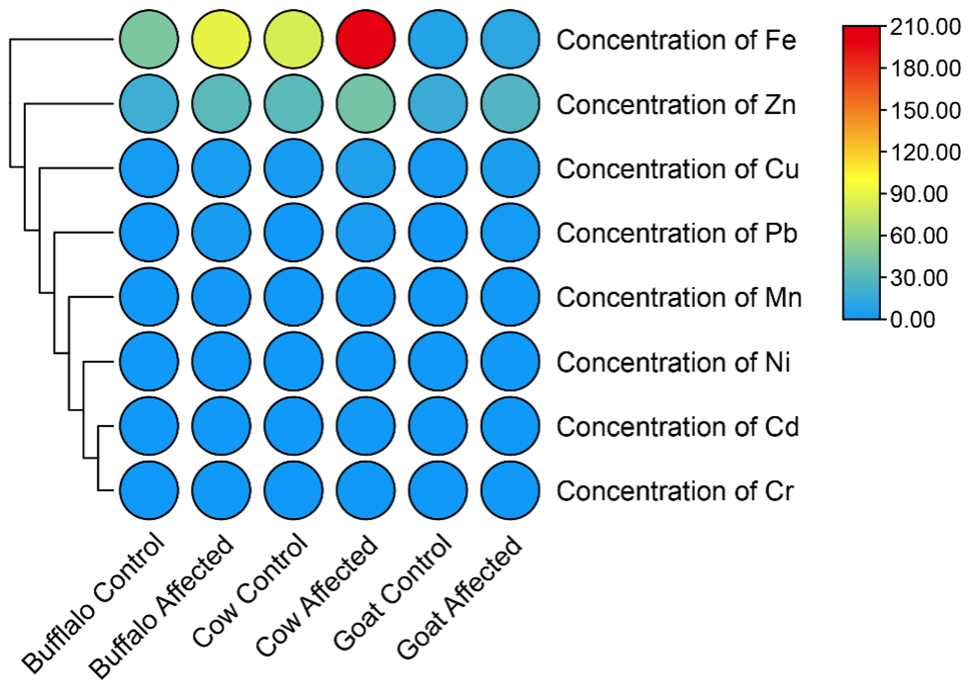
Concentration of Fe, Cu, Mn, Ni, Cr, Cd, Pb, and Zn mg/kg in buffalo, cow, and goat meat of control and affected group.

During the investigation of the concentration of heavy metals, the results revealed varying levels of heavy metals across the three livestock species meat (Figure 3). Specifically, the highest concentration of Cd was observed in cow meat (0.43 mg/kg). For Cu, cows exhibited the highest concentration (6.7 mg/kg), followed by buffaloes (4 mg/kg). Among the heavy metals tested, Fe demonstrated the most pronounced differences, with cows exhibiting the highest concentration (200 mg/kg) compared to buffaloes (90 mg/kg) and goats (12.07 mg/kg). Similar trends were observed for Mn, Ni, Pb, and Zn, with varying concentrations across the three species. These findings shed light on the differential accumulation of heavy metals in the meat of buffaloes, cows, and goats, emphasizing the importance of species-specific monitoring in mitigating potential health risks associated with heavy metal exposure through dairy consumption.

### Concentration of heavy metal (mg/kg) in buffalo, cow and goat milk

**Figure 4:**
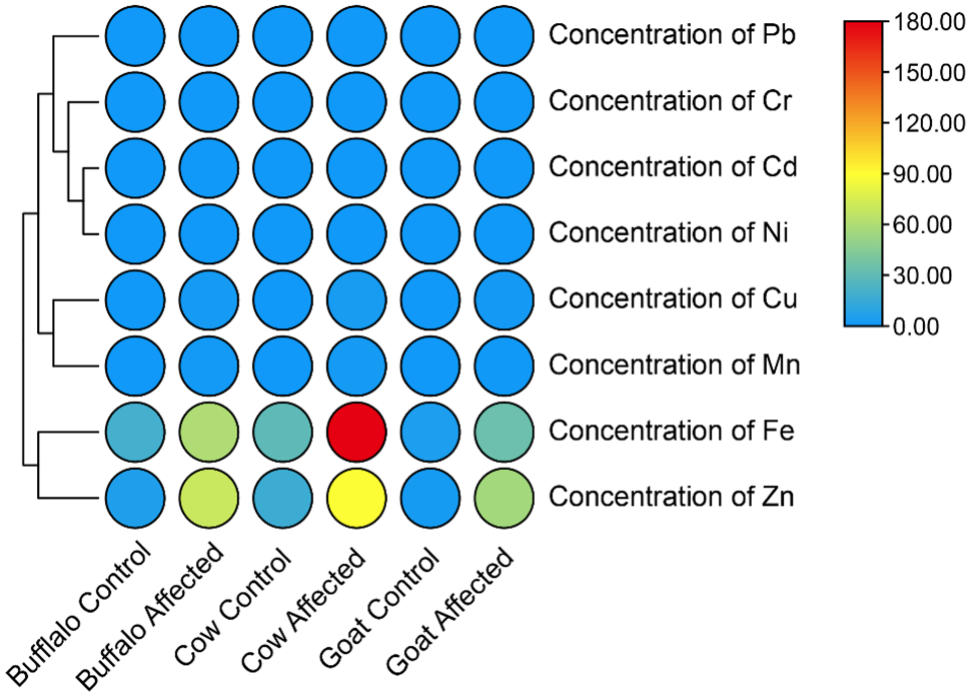
Concentration of Fe, Cu, Mn, Ni, Cr, Cd, Pb and Zn mg/kg in buffalo, cow and goat milk of control and affected group.

During the investigation, the concentration of heavy metals revealed varying levels across the three livestock species' milk (Figure 4). Affected animals had significantly higher heavy metal concentrations compared to the control groups. For instance, in the case of Copper (Cu), cows and goats had much higher concentrations (3 and 1.6 mg/kg, respectively). Similarly, in the case of Iron (Fe), cows had a much higher concentration (180 mg/kg) of heavy metal than all other species. These results suggested potential heavy metal contamination in the affected animal groups, which could have had adverse health implications.

## Discussion

The present study revealed that soil is contaminated with some heavy metals Fe, Cd, which have higher mean concentrations 6.96, 1.09 mg kg^−1^ and crossed the threshold^32^ value set by FAO/WHO 2007, respectively. The higher content in affected soil compared to control was most likely due to wastewater containing a higher concentration of metals. Waste water application builds up the metal concentration Fe, Pb, Zn in surface soil 0-15 cm. Soil irrigated with sewage from the city had higher content in some metals Fe, Cd, Cr. The level of Cd in soil irrigated with city effluent exceeded the standard guidelines set by FAO/WHO 2000^33^. Cereals, fruits, and vegetables are the main sources of heavy metals in the human body. The concentration of heavy metals in food depends on both the mobility and bioavailability of heavy metals in soil. Trace elements are vital in humans, plants, and animals' chemical, biological, and biochemical reactions. The concentration of heavy metals in food increases due to androgenic sources^34,35^. The higher concentration of analyzed metal in different cereals, wheat, and rice grown near the Hudiara drain might be due to irrigation of untreated industrial and domestic water that significantly affects the metal content in crops. Most metals' concentrations were above the international organizations' threshold limits. The highest concentration of Cu and Cd was reported in wheat and rice in most of the studies on soil receiving a high load of sewage sludge or wastewater^36^. Ni in sampled wheat grains irrigated through the Hudiara drain of the current study was higher than the permissible limits of FAO and WHO 2000^37^. The higher content of Ni in wheat grain might be due to different inputs of waste from different industries, especially glass, paint, and textile^38^. Cr content was higher than the established permissible limits from the different international organizations and potentially threatened people using affected wheat grains^39^. The observed level of Cd in cabbage is higher than the permissible limits of FAO, 2007. This might be due to the area's higher continuous wastewater usage^40^.

Leafy vegetables irrigated through industrial sewage water showed higher levels than the permissible limits^41,42^. Fe has significant importance for all crops regarding photosynthesis and other metabolites. The results revealed the Fe level is higher in leafy vegetables i.e., Brassica, cauliflower cabbage, cauliflower, and beets grown on affected/polluted soil than control/unpolluted soil. Our findings align with studies conducted on effluent industries^43^. Similarly, the mean concentration of Mn is in the range of previous studies conducted in Pakistan^43^. Pb concentration in the sampled vegetables aligns with studies conducted on effluent industries^43^. The findings of the conducted study regarding Zn concentration treated with Hudiara drain water and underground water are within permissible limits established by FAO and WHO, 2000^44^.

In this study, the leafy vegetables i.e., Cabbage, cauliflower, spinach, brassica, and turnip were studied for metal acquisition under irrigation through the Hudiara drain and underground water. The results revealed that bio-available concentrations of heavy metals were higher in vegetables irrigated with sewage water than underground water. The metals Pb, Cd, and Cr accumulated in leafy vegetables brassica, cabbage, cauliflower, and turnip have a higher value than permissible limits 0.3, 0.3, and 1.3 mg kg^−1^. This might be due to the continuous irrigation of sewage wastewater from the Hudiara drain, the main root of heavy metals accumulation in soils^5,45–47^. Beside this, the higher concentration of Lead, Cadmium, and Chromium in leafy vegetables from the permissible limits might be due to the presence of various industries installed on the way of Hudiara drain, Steels, and Iron foundries, rolling mills, and continuous broken steel coated with Cd and Pb in the wastewater. Furthermore, it has been reported that soil had a higher content of heavy metals irrigated with wastewater^48^.

Heavy metals in different vegetables cultivated on wastewater in Bahawalpur that have a higher concentration of Pb, Cr, and Cd; and exceeded the permissible limits. However, the present research showed that higher concentrations of Ni, Zn, Fe, and Cu in leafy vegetables grown near Hudiara drain did not exceed the threshold value established by WHO, 2007^49^. The plants/vegetables treated with waste water have a higher content of heavy metals than plants treated with fresh water. The variation in heavy metals content in leafy and root vegetables directly depends upon the nature of plants, physicochemical properties of soil, as well as the environment and anthropogenic activities^49^.

In the present research study, metals analyzed in drinking water were examined as per standard guidelines of WHO/ FAO, 2007. The concentration of metals Zn, Fe, Mn, and Cu was within tolerable limits according to WHO, 2007, and Pakistan EPA standards^50^. However, drinking water quality exhibited elevated levels in metals of Pb, Cr, Ni, and Cd from WHO's standard guidelines. The high level of Ni in the drinking water might be due to ultramafic industrial activities and continuous seepage of sewage water from the Hudiara drain in the study area. Lead in drinking water exceeded the permissible limits accomplished by WHO. The raised lead concentration in the study area might be due to the usage of pesticides on agricultural land, and continuous application of Hudiara drains' sewage water may lead to metal leaching^51^. Previous work has also illustrated the elevated lead levels in Lahore, Jhang, and Vehari^52^. The present study revealed the variation in metal concentration in the meat of buffalo, cow, and goat in affected/polluted and controlled/non-polluted areas of Hudiara drain. The higher concentration of Cd in raw meat of buffalo grazed in affected/polluted areas compared to controlled/unpolluted. Areas and Cd concentrations in raw buffalo and cow meat exceeded the permissible limit set by the International Dairy Federation 1979. It is the most acceptable limit for Cd in raw meat^53^.

The Cd level in cow’s meat watering from the industrial effluent and drinking from the uncontaminated river water. They concluded that Cd level was twice as high in cow’s meat collected from the contaminated area than in the non-contaminated area. Iftikhar and his colleagues collected raw meat from urban and rural areas, illustrating that Cd level was higher in the urban areas than rural areas^54^. Lead in meat is one of the most toxic metals affecting human health. It is the most common industrial metal that can contaminate the soil, water, air, forage, and food^55^.

In the present study, the maximum mean lead level in cow’s meat was observed in the affected polluted areas, followed by buffalo and goat at 0.19, 0.16, and 0.12 mg kg^−1,^ respectively. However, the minimum Pb concentration in Goat meat was recorded in the controlled area 0.0003 mg kg^−1^. The higher metal level in meat could be due to the grazing of animals in polluted areas. The present study revealed lead concentration exceeded the permissible limits established by WHO^37^. The present study's findings align with the previous study, which reported a higher Pb level in the industrial polluted areas than in non-polluted areas. Similarly, the highest concentration was 23.24 mg L^−1^ of lead in cow’s meat; cows were drinking untreated sewage water in Faisalabad, Pakistan^56^.

In the present study, the highest mean level of nickel was observed in cows, followed by buffalo and goat 0.023, 0.018, and 0.012 mg L^−1^, respectively, reared in the contaminated areas; however, the low level was detected in the control or unpolluted area. Fe is an essential trace element that stimulates several metabolic reactions in the human body, although its higher level may produce toxicity and cause health issues. In the present study, a higher level of Fe was recorded in cow’s meat, followed by buffalo and goat meat, respectively, compared to the controlled area. It exceeded the permissible limits furnished by IDF 1979. A higher level of Fe in cow’s meat near the industrial polluted areas compared to the non-polluted areas in Egypt, and a significant level was also reported in India^52^. Copper has an essential role in human health; however, its higher level may cause various toxic effects on the human body. The level of Cu was recorded in the meat of cows followed by buffalo and goat, respectively, in polluted compared to non-polluted areas. The cow’s meat ranging from 0.0136 to 36 mg L^−1,^ and exceeds the permissible level 0.01 mg L^−1^ set by IDF, 1979 world., reported in various studies^53^. The current work revealed a higher level of Cr in raw meat of buffalo, cows, and goats grazing in the polluted areas compared to the controlled areas, an exceeded the permissible value set by IDF. However, its value is below what was detected in this study and reported in Codex Alimentarius Commission, 2011. All the detected values of Zn were within permissible limits of WHO 121 mg kg^−1^. The Zn concentration in camel, cattle, buffalo and goat meat from various regions of KPK. They reported the Zn concentration was within permissible limits and far below that of current work. The contaminated heavy metals in fodder crops are used as feed and contaminate animal feed^53^.

Excessive Cu causes disturbance in the immunity system, dermatitis, nervous system, and gastrointestinal diseases^52^ . The permissible concentration of Cu in meat is .01 µg/g IDF 1979. According to Puls 1994, the normal median value of Cu in meat is 0.5 µg/g. The daily intake of Co is 4.33 µg/kg/day and Cd 0.011 µg/kg/day. In the current research, the higher level of heavy metals in the raw meat of examined animals might be attributed to the use of sewage water of Hudiara drain for agricultural purposes and direct access to polluted water for drinking. Heavy metal toxicity caused by animal meat ingestion has created human health issues for many years^13^.

Overall, the findings of the present work showed a higher level of heavy metal contents in meat samples of affected/polluted areas compared to control/non-polluted areas. The result of the present study showed a higher level of Pb in cows 4.3 mg kg^−1^ followed by buffalo and goats 3.2 and 2 mg kg^−1,^ respectively; its level was above the permissible limits of 0.1 mg kg^−1^ set by WHO^13^. The prescribed level in the present work revealed that cadmium concentration in cow, goat, and buffalo is higher than the permissible limits.

In Iran, a similar trend was observed for Cd levels in meat, as its concentration exceeded the tolerable limits set by WHO in 2007^13^. Besides this, levels of Zn and Cu in the prescribed study revealed a difference in the level of metals detected in the affected as compared to the control; however, their level did not cross the acceptable limits set by WHO. The content of Zn and Cu was analyzed in meat samples collected from the Lahore Market. Cr and Ni concentrations^57^ in the meat of analyzed samples collected from the polluted and non-polluted areas showed variation in its level; although the higher level detected in the collected samples; was lower than the acceptable limits USDA, 2006. Fe concentration in collected samples of buffalo and goat are below the tolerable limits, however, the concentration in cow crossed the permissible limits. The variation in the level of Fe in meat might be due to the feeding behavior of respective animals^57^. The variation of Fe concentration was examined in the meat of both goat and cow. The results of the present study illustrated the significant variation in analyzed metals samples in human blood. The higher concentration was detected in samples collected from the vicinity of the Hudiara drain; however, the lower metal concentration was recorded in samples taken far away from the locality of the Hudiara drain. The levels of most metals content in human blood were higher than the permissible limits. The significant level of metals in the human population living near the sewage effluent^58^. Detected a potential threat of Pb, Ni, and Cd in human blood. The accumulation of heavy metals in the human body generates ROS and RNS, which are directly involved in damaging lipids, proteins, and DNA. The biochemical properties alter due to cross-linking of DNA. The adduct of MDA and 4-HNE also affects the synthesis of protein. It promotes cell death and activates the signaling pathway.

The excess formation of free radicals weak the antioxidant system, which causes oxidative stress and as result increases the process of lipid peroxidation, malondialdehyde MDA, Isoprostane Iso, and an adduct of DNA^59^. Studies have proved that 8-OHdG increases with exposure of toxic metals, as the association between 8-OHdG and cadmium is very strong. Malondialdehyde MDA can measure the damaging of free radicals. The malondialdehyde MDA can be measured by reaction with thiobarbituric acid using a spectrophotometer. In our study, heavy metals cause ROS and enhance MDA^60^.

The more effective marker of lipid peroxidation is Isoprostane, which arises from unsaturated fatty acids. The areas of high-level heavy metals will result in severe complications in the future. National and international cooperation should take major steps to control exposure to heavy metals by providing safe guidelines on using chemicals and industries. There should be proper channel lining of sewerage channels to avoid seepage of waste water and toxic chemicals in the soil of the adjacent area.

### Supplementary Information

Supplementary Table1.

Supplementary Table 2.

Supplementary Table 3.

Supplementary Table 4.

## Data Availability

The produced, collected, or generated during the study has been given in the manuscript file and its supplementary file.
