# Supplementary material for: DNA damage in inhabitants exposed to heavy metals near Hudiara drain, Lahore, Pakistan
Source: Sci Rep. 2024 Apr 10;14:8408. doi: 10.1038/s41598-024-58655-x (PMC11006874; doi:10.1038/s41598-024-58655-x)
Supplement: Supplementary file 1 — Supplementary Table1. [file 41598_2024_58655_MOESM1_ESM.docx]

**Supplementary Materials**

**Table 1. Demograpghic data for male and female**

| **S.NO** | **Weight (Kg)** | **Age (Years)** | **Gender** | **Smoking** | **Education** | **Working** |
| --- | --- | --- | --- | --- | --- | --- |
| 1 | 56 | 55 | MALE | Non-Smoker | Middle | Laborer |
| 2 | 60 | 49 | MALE | Non-Smoker | Secondary | Agricultural Laborer |
| 3 | 64 | 50 | MALE | Non-Smoker | Primary | Unskilled Worker |
| 4 | 72 | 74 | MALE | Non-Smoker | Primary | Street Vendor |
| 5 | 60 | 59 | MALE | Non-Smoker | Secondary | Custodial Worker |
| 6 | 78 | 54 | MALE | Non-Smoker | Middle | Farm Worker |
| 7 | 68 | 69 | MALE | Non-Smoker | Middle | Janitor |
| 8 | 62 | 57 | MALE | Non-Smoker | Primary | Waiter/Waitress |
| 9 | 60 | 56 | MALE | Non-Smoker | Secondary | Delivery Driver |
| 10 | 65 | 59 | MALE | Non-Smoker | Primary | Housekeeper |
| 11 | 53 | 62 | MALE | Non-Smoker | Primary | Security Guard |
| 12 | 64 | 79 | MALE | Non-Smoker | Middle | Street Vendor |
| 13 | 65 | 59 | MALE | Non-Smoker | Secondary | Construction Laborer |
| 14 | 69 | 64 | MALE | Non-Smoker | Secondary | Dishwasher |
| 15 | 60 | 69 | MALE | Non-Smoker | Middle | Custodial Worker |
| 16 | 59 | 49 | MALE | Non-Smoker | Primary | Retail Salesperson |
| 17 | 78 | 54 | MALE | Non-Smoker | Secondary | Factory Worker |
| 18 | 57 | 49 | MALE | Non-Smoker | Middle | Childcare Worker |
| 19 | 65 | 55 | MALE | Non-Smoker | Primary | Laborer |
| 20 | 62 | 64 | MALE | Non-Smoker | Secondary | Street Vendor |
| 21 | 60 | 63 | MALE | Non-Smoker | Middle | Laborer |
| 22 | 59 | 69 | MALE | Non-Smoker | Primary | Custodial Worker |
| 23 | 81 | 54 | MALE | Non-Smoker | Secondary | Agricultural Laborer |
| 24 | 72 | 54 | MALE | Non-Smoker | Middle | Unskilled Worker |
| 25 | 60 | 62 | MALE | Non-Smoker | Primary | Street Vendor |
| 26 | 62 | 49 | MALE | Non-Smoker | Secondary | Custodial Worker |
| 27 | 58 | 59 | MALE | Non-Smoker | Middle | Farm Worker |
| 28 | 59 | 64 | MALE | Non-Smoker | Secondary | Janitor |
| 29 | 60 | 49 | MALE | Non-Smoker | Secondary | Waiter |
| 30 | 69 | 59 | MALE | Non-Smoker | Secondary | Delivery Driver |
| 31 | 86 | 49 | MALE | Non-Smoker | Uneducated | Construction Laborer |
| 32 | 88 | 69 | MALE | Non-Smoker | Uneducated | Laborer |
| 33 | 97 | 39 | MALE | Non-Smoker | Uneducated | Street Vendor |
| 34 | 85 | 49 | MALE | Non-Smoker | Uneducated | Butcher |
| 35 | 76 | 49 | MALE | Non-Smoker | Uneducated | Shop Worker |
| 36 | 65 | 59 | MALE | Non-Smoker | Uneducated | Butcher |
| 37 | 70 | 64 | MALE | Non-Smoker | Uneducated | Milkman |
| 38 | 82 | 64 | MALE | Non-Smoker | Uneducated | Working in Field |
| 39 | 78 | 49 | MALE | Non-Smoker | Uneducated | Laborer |
| 40 | 72 | 54 | MALE | Non-Smoker | Uneducated | Street Vendor |
| 41 | 74 | 55 | MALE | Non-Smoker | Uneducated | Laborer |
| 42 | 69 | 84 | MALE | Non-Smoker | Uneducated | Unskilled Worker |
| 43 | 60 | 54 | MALE | Non-Smoker | Uneducated | Working in Field |
| 44 | 100 | 64 | MALE | Non-Smoker | Uneducated | Shop Worker |
| 45 | 86 | 70 | MALE | Non-Smoker | Uneducated | Butcher |
| 46 | 85 | 57 | MALE | Non-Smoker | Uneducated | Butcher |
| 47 | 80 | 58 | MALE | Non-Smoker | Uneducated | Peon |
| 48 | 74 | 55 | MALE | Non-Smoker | Uneducated | Butcher |
| 49 | 69 | 65 | MALE | Non-Smoker | Uneducated | Working in Field |
| 50 | 96 | 70 | MALE | Non-Smoker | Uneducated | Butcher |
| 51 | 69 | 65 | MALE | Non-Smoker | Uneducated | Working in Field |
| 52 | 96 | 69 | MALE | Non-Smoker | Uneducated | Working in Field |
| 53 | 85 | 70 | MALE | Non-Smoker | Uneducated | Working in Field |
| 54 | 76 | 65 | MALE | Non-Smoker | Uneducated | Butcher |
| 55 | 96 | 67 | MALE | Non-Smoker | Uneducated | Shop Worker |
| 56 | 87 | 68 | MALE | Non-Smoker | Uneducated | Street Vendor |
| 57 | 86 | 70 | MALE | Non-Smoker | Uneducated | Laborer |
| 58 | 96 | 69 | MALE | Non-Smoker | Uneducated | Laborer |
| 59 | 82 | 62 | MALE | Non-Smoker | Uneducated | Unskilled Worker |
| 60 | 96 | 67 | MALE | Non-Smoker | Uneducated | Working in Field |
| 61 | 62 | 60 | MALE | Non-Smoker | Uneducated | Peon |
| 62 | 86 | 40 | MALE | Non-Smoker | Uneducated | Shop Worker |
| 63 | 81 | 41 | MALE | Non-Smoker | Uneducated | Laborer |
| 64 | 66 | 28 | MALE | Non-Smoker | Uneducated | Unskilled Worker |
| 65 | 65 | 30 | MALE | Non-Smoker | Uneducated | Shop Worker |
| 66 | 86 | 34 | MALE | Non-Smoker | Uneducated | Laborer |
| 67 | 65 | 35 | MALE | Non-Smoker | Uneducated | Working in Field |
| 68 | 67 | 32 | MALE | Non-Smoker | Uneducated | Butcher |
| 69 | 70 | 35 | MALE | Non-Smoker | Uneducated | Shop Worker |
| 70 | 66 | 14 | MALE | Non-Smoker | Uneducated | Milkman |
| 71 | 68 | 41 | MALE | Non-Smoker | Uneducated | Shop Worker |
| 72 | 63 | 40 | MALE | Non-Smoker | Uneducated | Butcher |
| 73 | 64 | 39 | MALE | Non-Smoker | Uneducated | Working in Field |
| 74 | 89 | 42 | MALE | Non-Smoker | Uneducated | Shop Worker |
| 75 | 81 | 51 | MALE | Non-Smoker | Uneducated | Butcher |
| 76 | 83 | 40 | MALE | Non-Smoker | Uneducated | Street Vendor |
| 77 | 86 | 39 | MALE | Non-Smoker | Uneducated | Shop Worker |
| 78 | 65 | 42 | MALE | Non-Smoker | Uneducated | Laborer |
| 79 | 69 | 41 | MALE | Non-Smoker | Uneducated | Butcher |
| 80 | 68 | 39 | MALE | Non-Smoker | Uneducated | Unskilled Worker |
| 81 | 72 | 41 | MALE | Non-Smoker | Uneducated | Butcher |
| 82 | 71 | 40 | MALE | Non-Smoker | Uneducated | Shop Worker |
| 83 | 66 | 44 | MALE | Non-Smoker | Uneducated | Street Vendor |
| 84 | 43 | 45 | MALE | Non-Smoker | Uneducated | Butcher |
| 85 | 51 | 42 | MALE | Non-Smoker | Uneducated | Shop Worker |
| 86 | 57 | 44 | MALE | Non-Smoker | Uneducated | Butcher |
| 87 | 59 | 46 | MALE | Non-Smoker | Uneducated | Working in Field |
| 88 | 55 | 47 | MALE | Non-Smoker | Uneducated | Working in Field |
| 89 | 72 | 40 | MALE | Non-Smoker | Uneducated | Milkman |
| 90 | 53 | 34 | MALE | Non-Smoker | Uneducated | Milkman |
| 91 | 54 | 25 | MALE | Non-Smoker | Uneducated | Shop Worker |
| 92 | 66 | 26 | MALE | Non-Smoker | Uneducated | Laborer |
| 93 | 68 | 76 | MALE | Non-Smoker | Uneducated | Shop Worker |
| 94 | 69 | 41 | MALE | Non-Smoker | Uneducated | Working in Field |
| 95 | 95 | 51 | MALE | Non-Smoker | Uneducated | Butcher |
| 96 | 53 | 51 | MALE | Non-Smoker | Uneducated | Shop Worker |
| 97 | 64 | 52 | MALE | Non-Smoker | Uneducated | Laborer |
| 98 | 54 | 55 | MALE | Non-Smoker | Uneducated | Butcher |
| 99 | 67 | 47 | MALE | Non-Smoker | Uneducated | Shop Worker |
| 100 | 46 | 44 | MALE | Non-Smoker | Uneducated | Street Vendor |
| 101 | 67 | 47 | MALE | Non-Smoker | Uneducated | Laborer |
| 102 | 69 | 70 | MALE | Non-Smoker | Uneducated | Street Vendor |
| 103 | 68 | 80 | MALE | Non-Smoker | Uneducated | Butcher |
| 104 | 59 | 55 | MALE | Non-Smoker | Uneducated | Laborer |
| 105 | 45 | 42 | MALE | Non-Smoker | Uneducated | Butcher |
| 106 | 67 | 32 | MALE | Non-Smoker | Uneducated | Butcher |
| 107 | 66 | 22 | MALE | Non-Smoker | Uneducated | Milkman |
| 108 | 69 | 25 | MALE | Non-Smoker | Uneducated | Shop Worker |
| 109 | 70 | 24 | MALE | Non-Smoker | Uneducated | Street Vendor |
| 110 | 71 | 25 | MALE | Non-Smoker | Uneducated | Butcher |
| 111 | 51 | 52 | MALE | Non-Smoker | Uneducated | Street Vendor |
| 112 | 55 | 42 | MALE | Non-Smoker | Uneducated | Butcher |
| 113 | 67 | 25 | MALE | Non-Smoker | Uneducated | Laborer |
| 114 | 52 | 24 | MALE | Non-Smoker | Uneducated | Butcher |
| 115 | 53 | 25 | MALE | Non-Smoker | Uneducated | Milkman |
| 116 | 54 | 25 | MALE | Non-Smoker | Uneducated | Street Vendor |
| 117 | 67 | 26 | MALE | Non-Smoker | Uneducated | Butcher |
| 118 | 59 | 25 | MALE | Non-Smoker | Uneducated | Milkman |
| 119 | 57 | 24 | MALE | Non-Smoker | Uneducated | Butcher |
| 120 | 59 | 27 | MALE | Non-Smoker | Uneducated | Laborer |
| 121 | 60 | 27 | MALE | Non-Smoker | Uneducated | Street Vendor |
| 122 | 58 | 28 | MALE | Non-Smoker | Uneducated | Butcher |
| 123 | 59 | 27 | MALE | Non-Smoker | Uneducated | Shop Worker |
| 124 | 67 | 26 | MALE | Non-Smoker | Uneducated | Butcher |
| 125 | 56 | 25 | MALE | Non-Smoker | Uneducated | Milkman |
| 126 | 59 | 25 | MALE | Non-Smoker | Uneducated | Working in Field |
| 127 | 60 | 24 | MALE | Non-Smoker | Uneducated | Laborer |
| 128 | 89 | 40 | MALE | Non-Smoker | Uneducated | Street Vendor |
| 129 | 78 | 39 | MALE | Non-Smoker | Uneducated | Laborer |
| 130 | 59 | 24 | MALE | Non-Smoker | Uneducated | Unskilled Worker |
| 131 | 69 | 55 | MALE | Non-Smoker | Uneducated | Working in Field |
| 132 | 71 | 42 | MALE | Non-Smoker | Uneducated | Shop Worker |
| 133 | 66 | 49 | MALE | Non-Smoker | Uneducated | Butcher |
| 134 | 46 | 50 | MALE | Non-Smoker | Uneducated | Butcher |
| 135 | 53 | 42 | MALE | Non-Smoker | Uneducated | Peon |
| 136 | 66 | 41 | MALE | Non-Smoker | Uneducated | Butcher |
| 137 | 59 | 39 | MALE | Non-Smoker | Uneducated | Working in Field |
| 138 | 66 | 47 | MALE | Non-Smoker | Uneducated | Butcher |
| 139 | 67 | 45 | MALE | Non-Smoker | Uneducated | Working in Field |
| 140 | 70 | 46 | MALE | Non-Smoker | Uneducated | Working in Field |
| 141 | 71 | 44 | MALE | Non-Smoker | Uneducated | Working in Field |
| 142 | 66 | 40 | MALE | Non-Smoker | Uneducated | Butcher |
| 143 | 59 | 41 | MALE | Non-Smoker | Uneducated | Shop Worker |
| 144 | 52 | 42 | MALE | Non-Smoker | Uneducated | Street Vendor |
| 145 | 55 | 45 | MALE | Non-Smoker | Uneducated | Laborer |
| 146 | 66 | 24 | MALE | Non-Smoker | Uneducated | Laborer |
| 147 | 59 | 23 | MALE | Non-Smoker | Uneducated | Unskilled Worker |
| 148 | 56 | 39 | MALE | Non-Smoker | Uneducated | Working in Field |
| 149 | 70 | 24 | MALE | Non-Smoker | Uneducated | Peon |
| 150 | 86 | 34 | MALE | Non-Smoker | Uneducated | Shop Worker |
| 151 | 87 | 40 | MALE | Non-Smoker | Uneducated | Laborer |
| 152 | 52 | 24 | MALE | Non-Smoker | Uneducated | Unskilled Worker |
| 153 | 53 | 55 | MALE | Non-Smoker | Uneducated | Shop Worker |
| 154 | 54 | 40 | MALE | Non-Smoker | Uneducated | Laborer |
| 155 | 55 | 57 | MALE | Non-Smoker | Uneducated | Working in Field |
| 156 | 66 | 55 | MALE | Non-Smoker | Uneducated | Butcher |
| 157 | 51 | 53 | MALE | Non-Smoker | Uneducated | Shop Worker |
| 158 | 52 | 41 | MALE | Non-Smoker | Uneducated | Milkman |
| 159 | 42 | 24 | MALE | Non-Smoker | Uneducated | Shop Worker |
| 160 | 42 | 27 | MALE | Non-Smoker | Uneducated | Butcher |
| 161 | 54 | 27 | MALE | Non-Smoker | Uneducated | Working in Field |
| 162 | 57 | 26 | MALE | Non-Smoker | Uneducated | Shop Worker |
| 163 | 43 | 14 | MALE | Non-Smoker | Uneducated | Laborer |
| 164 | 53 | 25 | MALE | Non-Smoker | Uneducated | Street Vendor |
| 165 | 54 | 66 | MALE | Non-Smoker | Uneducated | Butcher |
| 166 | 67 | 25 | MALE | Non-Smoker | Uneducated | Shop Worker |
| 167 | 42 | 24 | MALE | Non-Smoker | Uneducated | Butcher |
| 168 | 41 | 25 | MALE | Non-Smoker | Uneducated | Milkman |
| 169 | 43 | 25 | MALE | Non-Smoker | Uneducated | Working in Field |
| 170 | 34 | 55 | MALE | Non-Smoker | Uneducated | Laborer |
| **171** | **54** | **51** | **FEMALE** | **Non-Smoker** | **Middle** | **Office Manager** |
| 172 | 63 | 44 | FEMALE | Non-Smoker | Secondary | Retail Salesperson |
| 173 | 62 | 66 | FEMALE | Non-Smoker | Primary | Housewife |
| 174 | 53 | 51 | FEMALE | Non-Smoker | Middle | Office Manager |
| 175 | 42 | 42 | FEMALE | Non-Smoker | Secondary | Retail Salesperson |
| 176 | 54 | 25 | FEMALE | Non-Smoker | Primary | Housewife |
| 177 | 52 | 24 | FEMALE | Non-Smoker | Secondary | Retail Salesperson |
| 178 | 57 | 35 | FEMALE | Non-Smoker | Middle | Teacher |
| 179 | 55 | 31 | FEMALE | Non-Smoker | Primary | Housewife |
| 180 | 59 | 41 | FEMALE | Non-Smoker | Secondary | Retail Salesperson |
| 181 | 57 | 45 | FEMALE | Non-Smoker | Middle | Office Manager |
| 182 | 55 | 66 | FEMALE | Non-Smoker | Primary | Housewife |
| 183 | 53 | 41 | FEMALE | Non-Smoker | Secondary | Retail Salesperson |
| 184 | 54 | 40 | FEMALE | Non-Smoker | Primary | Housewife |
| 185 | 64 | 26 | FEMALE | Non-Smoker | Middle | Teacher |
| 186 | 62 | 25 | FEMALE | Non-Smoker | Primary | Housewife |
| 187 | 61 | 41 | FEMALE | Non-Smoker | Secondary | Retail Salesperson |
| 188 | 55 | 40 | FEMALE | Non-Smoker | Middle | Office Manager |
| 189 | 66 | 23 | FEMALE | Non-Smoker | Secondary | Retail Salesperson |
| 190 | 69 | 25 | FEMALE | Non-Smoker | Primary | Housewife |
| 191 | 55 | 26 | FEMALE | Non-Smoker | Middle | Teacher |
| 192 | 82 | 35 | FEMALE | Non-Smoker | Secondary | Retail Salesperson |
| 193 | 52 | 35 | FEMALE | Non-Smoker | Primary | Housewife |
| 194 | 71 | 26 | FEMALE | Non-Smoker | Middle | Teacher |
| 195 | 66 | 35 | FEMALE | Non-Smoker | Secondary | Retail Salesperson |
| 196 | 35 | 25 | FEMALE | Non-Smoker | Primary | Housewife |
| 197 | 86 | 26 | FEMALE | Non-Smoker | Secondary | Retail Salesperson |
| 198 | 71 | 41 | FEMALE | Non-Smoker | Middle | Office Manager |
| 199 | 57 | 26 | FEMALE | Non-Smoker | Primary | Housewife |
| 200 | 86 | 35 | FEMALE | Non-Smoker | Secondary | Retail Salesperson |
| 201 | 71 | 25 | FEMALE | Non-Smoker | Uneducated | Housewife |
| 202 | 69 | 26 | FEMALE | Non-Smoker | Uneducated | Housewife |
| 203 | 72 | 25 | FEMALE | Non-Smoker | Uneducated | Housekeeper |
| 204 | 66 | 28 | FEMALE | Non-Smoker | Uneducated | Cleaner |
| 205 | 69 | 24 | FEMALE | Non-Smoker | Uneducated | Nanny |
| 206 | 66 | 26 | FEMALE | Non-Smoker | Uneducated | Domestic Helper |
| 207 | 69 | 25 | FEMALE | Non-Smoker | Uneducated | Farmer |
| 208 | 65 | 26 | FEMALE | Non-Smoker | Uneducated | Laborer |
| 209 | 66 | 25 | FEMALE | Non-Smoker | Uneducated | Street Vendor |
| 210 | 62 | 28 | FEMALE | Non-Smoker | Uneducated | Beggar |
| 211 | 63 | 26 | FEMALE | Non-Smoker | Uneducated | Seamstress |
| 212 | 85 | 23 | FEMALE | Non-Smoker | Uneducated | Maid |
| 213 | 81 | 25 | FEMALE | Non-Smoker | Uneducated | Caretaker |
| 214 | 55 | 66 | FEMALE | Non-Smoker | Uneducated | Retired |
| 215 | 67 | 51 | FEMALE | Non-Smoker | Uneducated | Housewife |
| 216 | 55 | 45 | FEMALE | Non-Smoker | Uneducated | Housewife |
| 217 | 58 | 40 | FEMALE | Non-Smoker | Uneducated | Housewife |
| 218 | 59 | 25 | FEMALE | Non-Smoker | Uneducated | Housekeeper |
| 219 | 67 | 46 | FEMALE | Non-Smoker | Uneducated | Street Vendor |
| 220 | 55 | 41 | FEMALE | Non-Smoker | Uneducated | Cleaner |
| 221 | 53 | 26 | FEMALE | Non-Smoker | Uneducated | Nanny |
| 222 | 64 | 42 | FEMALE | Non-Smoker | Uneducated | Domestic Helper |
| 223 | 66 | 40 | FEMALE | Non-Smoker | Uneducated | Farmer |
| 224 | 56 | 26 | FEMALE | Non-Smoker | Uneducated | Laborer |
| 225 | 51 | 25 | FEMALE | Non-Smoker | Uneducated | Street Vendor |
| 226 | 52 | 46 | FEMALE | Non-Smoker | Uneducated | Beggar |
| 227 | 55 | 24 | FEMALE | Non-Smoker | Uneducated | Seamstress |
| 228 | 57 | 25 | FEMALE | Non-Smoker | Uneducated | Maid |
| 229 | 59 | 55 | FEMALE | Non-Smoker | Uneducated | Retired |
| 230 | 58 | 50 | FEMALE | Non-Smoker | Uneducated | Housewife |
| 231 | 59 | 51 | FEMALE | Non-Smoker | Uneducated | Housewife |
| 232 | 52 | 51 | FEMALE | Non-Smoker | Uneducated | Housewife |
| 233 | 55 | 50 | FEMALE | Non-Smoker | Uneducated | Housewife |
| 234 | 59 | 53 | FEMALE | Non-Smoker | Uneducated | Housewife |
| 235 | 57 | 56 | FEMALE | Non-Smoker | Uneducated | Housewife |
| 236 | 59 | 46 | FEMALE | Non-Smoker | Uneducated | Street Vendor |
| 237 | 60 | 47 | FEMALE | Non-Smoker | Uneducated | Beggar |
| 238 | 52 | 48 | FEMALE | Non-Smoker | Uneducated | Cleaner |
| 239 | 59 | 15 | FEMALE | Non-Smoker | Uneducated | Child Laborer |
| 240 | 43 | 42 | FEMALE | Non-Smoker | Uneducated | Housewife |
| 241 | 54 | 25 | FEMALE | Non-Smoker | Uneducated | Housekeeper |
| 242 | 66 | 26 | FEMALE | Non-Smoker | Uneducated | Nanny |
| 243 | 59 | 25 | FEMALE | Non-Smoker | Uneducated | Street Vendor |
| 244 | 55 | 41 | FEMALE | Non-Smoker | Uneducated | Cleaner |
| 245 | 76 | 25 | FEMALE | Non-Smoker | Uneducated | Laborer |
| 246 | 73 | 26 | FEMALE | Non-Smoker | Uneducated | Domestic Helper |
| 247 | 53 | 25 | FEMALE | Non-Smoker | Uneducated | Farmer |
| 248 | 66 | 25 | FEMALE | Non-Smoker | Uneducated | Beggar |
| 249 | 55 | 24 | FEMALE | Non-Smoker | Uneducated | Maid |
| 250 | 52 | 26 | FEMALE | Non-Smoker | Uneducated | Retired |
| 251 | 55 | 25 | FEMALE | Non-Smoker | Uneducated | Seamstress |
| 252 | 51 | 24 | FEMALE | Non-Smoker | Uneducated | Housewife |
| 253 | 54 | 24 | FEMALE | Non-Smoker | Uneducated | Housewife |
| 254 | 53 | 21 | FEMALE | Non-Smoker | Uneducated | Housewife |
| 255 | 55 | 24 | FEMALE | Non-Smoker | Uneducated | Street Vendor |
| 256 | 46 | 42 | FEMALE | Non-Smoker | Uneducated | Beggar |
| 257 | 47 | 40 | FEMALE | Non-Smoker | Uneducated | Cleaner |
| 258 | 46 | 25 | FEMALE | Non-Smoker | Uneducated | Laborer |
| 259 | 49 | 40 | FEMALE | Non-Smoker | Uneducated | Domestic Helper |
| 260 | 48 | 42 | FEMALE | Non-Smoker | Uneducated | Street Vendor |
| 261 | 44 | 46 | FEMALE | Non-Smoker | Uneducated | Beggar |
| 262 | 53 | 45 | FEMALE | Non-Smoker | Uneducated | Housewife |
| 263 | 46 | 48 | FEMALE | Non-Smoker | Uneducated | Housewife |
| 264 | 54 | 47 | FEMALE | Non-Smoker | Uneducated | Farmer |
| 265 | 57 | 49 | FEMALE | Non-Smoker | Uneducated | Laborer |
| 266 | 58 | 48 | FEMALE | Non-Smoker | Uneducated | Maid |
| 267 | 59 | 45 | FEMALE | Non-Smoker | Uneducated | Domestic Helper |
| 268 | 55 | 41 | FEMALE | Non-Smoker | Uneducated | Cleaner |
| 269 | 57 | 43 | FEMALE | Non-Smoker | Uneducated | Beggar |
| 270 | 60 | 40 | FEMALE | Non-Smoker | Uneducated | Street Vendor |
| 271 | 59 | 41 | FEMALE | Non-Smoker | Uneducated | Cleaner |
| 272 | 58 | 45 | FEMALE | Non-Smoker | Uneducated | Street Sweeper |
| 273 | 57 | 46 | FEMALE | Non-Smoker | Uneducated | Beggar |
| 274 | 60 | 45 | FEMALE | Non-Smoker | Uneducated | Beggar |
| 275 | 58 | 42 | FEMALE | Non-Smoker | Uneducated | Cleaner |
| 276 | 57 | 43 | FEMALE | Non-Smoker | Uneducated | Domestic Helper |
| 277 | 59 | 46 | FEMALE | Non-Smoker | Uneducated | Street Vendor |
| 278 | 55 | 41 | FEMALE | Non-Smoker | Uneducated | Beggar |
| 279 | 41 | 47 | FEMALE | Non-Smoker | Uneducated | Street Sweeper |
| 280 | 44 | 48 | FEMALE | Non-Smoker | Uneducated | Laborer |
| 281 | 41 | 49 | FEMALE | Non-Smoker | Uneducated | Cleaner |
| 282 | 41 | 45 | FEMALE | Non-Smoker | Uneducated | Street Vendor |
| 283 | 46 | 48 | FEMALE | Non-Smoker | Uneducated | Beggar |
| 284 | 42 | 47 | FEMALE | Non-Smoker | Uneducated | Street Sweeper |
| 285 | 46 | 45 | FEMALE | Non-Smoker | Uneducated | Cleaner |
| 286 | 41 | 48 | FEMALE | Non-Smoker | Uneducated | Domestic Helper |
| 287 | 53 | 42 | FEMALE | Non-Smoker | Uneducated | Laborer |
| 288 | 41 | 40 | FEMALE | Non-Smoker | Uneducated | Street Vendor |
| 289 | 48 | 48 | FEMALE | Non-Smoker | Uneducated | Beggar |
| 290 | 61 | 30 | FEMALE | Non-Smoker | Uneducated | Child Laborer |
| 291 | 43 | 57 | FEMALE | Non-Smoker | Uneducated | Cleaner |
| 292 | 51 | 29 | FEMALE | Non-Smoker | Uneducated | Retired |
| 293 | 42 | 47 | FEMALE | Non-Smoker | Uneducated | Street Sweeper |
| 294 | 45 | 32 | FEMALE | Non-Smoker | Uneducated | Cleaner |
| 295 | 61 | 39 | FEMALE | Non-Smoker | Uneducated | Domestic Helper |
| 296 | 48 | 39 | FEMALE | Non-Smoker | Uneducated | Laborer |
| 297 | 54 | 53 | FEMALE | Non-Smoker | Uneducated | Street Vendor |
| 298 | 43 | 70 | FEMALE | Non-Smoker | Uneducated | Beggar |
| 299 | 53 | 67 | FEMALE | Non-Smoker | Uneducated | Beggar |
| 300 | 42 | 53 | FEMALE | Non-Smoker | Uneducated | Street Sweeper |
